# Proteolytic activation of the epithelial sodium channel (ENaC) by factor VII activating protease (FSAP) and its relevance for sodium retention in nephrotic mice

**DOI:** 10.1007/s00424-021-02639-7

**Published:** 2021-12-06

**Authors:** Ferruh Artunc, Bernhard N. Bohnert, Jonas C. Schneider, Tobias Staudner, Florian Sure, Alexandr V. Ilyaskin, Matthias Wörn, Daniel Essigke, Andrea Janessa, Nis V. Nielsen, Andreas L. Birkenfeld, Michael Etscheid, Silke Haerteis, Christoph Korbmacher, Sandip M. Kanse

**Affiliations:** 1 Department of Internal Medicine, Division of Endocrinology, Diabetology and Nephrology, University Hospital Tübingen, Tubingen, Germany; 2 Institute of Diabetes Research and Metabolic Diseases (IDM) of the Helmholtz Center Munich at the University Tübingen, Tubingen, Germany; 3 German Center for Diabetes Research (DZD) at the University Tübingen, Tubingen, Germany; 4 Institute of Cellular and Molecular Physiology, Friedrich-Alexander University Erlangen-Nürnberg, Erlangen, Germany; 5 Institute of Basic Medical Sciences, University of Oslo, Oslo, Norway; 6 Paul Ehrlich Institute, Langen, Germany; 7 Institute of Anatomy, University of Regensburg, Regensburg, Germany

**Keywords:** Factor VII activating protease, FSAP-HABP2, Serine protease, Epithelial sodium channel (ENaC), Nephrotic syndrome

## Abstract

Proteolytic activation of the epithelial sodium channel (ENaC) by aberrantly filtered serine proteases is thought to contribute to renal sodium retention in nephrotic syndrome. However, the identity of the responsible proteases remains elusive. This study evaluated factor VII activating protease (FSAP) as a candidate in this context. We analyzed FSAP in the urine of patients with nephrotic syndrome and nephrotic mice and investigated its ability to activate human ENaC expressed in *Xenopus laevis* oocytes. Moreover, we studied sodium retention in FSAP-deficient mice (*Habp2*^−/−^) with experimental nephrotic syndrome induced by doxorubicin. In urine samples from nephrotic humans, high concentrations of FSAP were detected both as zymogen and in its active state. Recombinant serine protease domain of FSAP stimulated ENaC-mediated whole-cell currents in a time- and concentration-dependent manner. Mutating the putative prostasin cleavage site in γ-ENaC (γRKRK178AAAA) prevented channel stimulation by the serine protease domain of FSAP. In a mouse model for nephrotic syndrome, active FSAP was present in nephrotic urine of *Habp2*^+/+^ but not of *Habp2*^−/−^ mice. However, *Habp2*^−/−^ mice were not protected from sodium retention compared to nephrotic *Habp2*^+/+^ mice. Western blot analysis revealed that in nephrotic *Habp2*^−/−^ mice, proteolytic cleavage of α- and γ-ENaC was similar to that in nephrotic *Habp2*^+/+^ animals. In conclusion, active FSAP is excreted in the urine of nephrotic patients and mice and activates ENaC in vitro involving the putative prostasin cleavage site of γ-ENaC. However, endogenous FSAP is not essential for sodium retention in nephrotic mice.

## Introduction

Proteinuria, sodium retention and edema are hallmarks of patients with acute nephrotic syndrome. Considerable evidence has emerged that aberrantly filtered serine proteases resulting in proteasuria mediate sodium retention in nephrotic syndrome by proteolytically activating the epithelial sodium channel (ENaC) expressed in the distal tubule [2, 20, 25, 31, 35]. This concept is supported by our recent finding that the cleavage products of α- and γ-ENaC were upregulated in mice with experimental nephrotic syndrome [7]. Moreover, treatment with the serine protease inhibitor aprotinin prevented proteolytic ENaC activation and sodium retention as did the ENaC blocker amiloride [4, 7, 8, 40]. Currently, the exact identity of the essential serine proteases in experimental nephrotic syndrome remains unknown. We have demonstrated that the lack of urokinase plasminogen activator (*Plau*), plasmin (P*lg*), or plasma kallikrein (*Klkb1*) — all of which are aprotinin-sensitive — did not protect from sodium retention in experimental nephrotic syndrome of mice with genetic deletion of these proteins [4, 19, 40].

In search of other relevant serine proteases, we identified factor VII activating protease (FSAP) as a highly abundant serine protease in nephrotic urine samples by a proteomic approach [39]. In urine samples from nephrotic humans and mice, FSAP was found to be the second and third most abundant active serine protease, respectively [39]. FSAP, encoded by the *HABP2* gene, is a trypsin-like serine protease that is released by the liver and sensitive to aprotinin. It has a molecular weight of 64 kDa and therefore is not expected to undergo glomerular filtration in the healthy kidney. Histones that are released upon tissue damage, inflammation, or NETosis, and represent a response to injury signal, activate the zymogen form of FSAP in the circulation [41]. Activation is due to auto-cleavage of the single chain FSAP at position 313 which causes the formation of the active two chain form. Both chains are linked by an inter-molecular disulfide bond. Once FSAP is active in the circulation, it can cleave proteins from the hemostasis and complement system as well as cellular regulators such as growth factors and receptors of the protease-activated receptor family (PARs) [11]. Analysis of substrate preference of FSAP indicates a predilection for clusters of positively charged amino acids [24]. A naturally occurring single-nucleotide polymorphism in the FSAP-encoding gene (Marburg I, MI) leads to a replacement of one amino acid (G534E) in the protease domain and completely diminishes the proteolytic activity of FSAP [15]. In the plasma, the activity of FSAP is tightly controlled by a number of protease inhibitors, forming a complex with FSAP. These inhibitors include plasminogen activator inhibitor-1 (PAI-1), C1 esterase inhibitor, α2-antiplasmin, and last but not least antithrombin [44].

*Habp2*^−/−^ mice show no marked phenotype in unchallenged conditions but show enhanced liver fibrosis, neointimal formation, infarction after ischemic stroke, and were protected against thrombosis [11]. In these models, the lack of endogenous FSAP exacerbates the injury response and this is associated with enhanced inflammation.

So far, urinary excretion of FSAP in nephrotic syndrome has not yet been investigated. Also, it is unknown whether FSAP is able to cleave the γ-subunit of ENaC for proteolytic channel activation or is involved in the pathogenesis of the nephrotic syndrome. In the present investigation, we aimed to investigate the contribution of FSAP to ENaC-mediated sodium retention in nephrotic syndrome. We analyzed urine samples from nephrotic patients and investigated the ability of FSAP to activate human ENaC heterologously expressed in *Xenopus laevis* oocytes. Finally, we studied sodium retention in FSAP-deficient mice (*Habp2*^−/−^) with experimental nephrotic syndrome.

## Materials and methods

### Patient samples

Spot urine samples were derived from patients presenting to the University Hospital Tuebingen with acute nephrotic syndrome of various etiology. These samples had been included in one previous study [39]. Patients were included after they provided written informed consent. The study was approved by the local ethics committee of the University of Tuebingen (259/2012MPG23).

### Mouse studies

Experiments were performed on 3-month-old wildtype and FSAP deficient (*Habp2*^−/−^) mice generated as previously described [9, 38]. C57/BL6-*Habp2*^−/−^ mice were backcrossed onto a 129 S1/SvImJ background to confer susceptibility to experimental nephrotic syndrome [1, 3, 5]. Genotyping was done using PCR with the primers 5′-GTG TTC CGT GTC CTG CTG CTA ATC GCC CTG-3′ (wildtype), 5′-GAC GAA TTC ATG GAG GCT TTG CCA CAG AGT TC-3′ (common), and 5′-GCA GCG CAT CGC CTT CTA TCG CCT TCT TGA C-3′ (ko). Mice were kept on a 12:12-h light–dark cycle and fed a standard diet (ssniff, sodium content 0.24% corresponding to 104 µmol g^−1^, Soest, Germany) with tap water ad libitum. Experimental nephrotic syndrome was induced after a single intravenous injection of doxorubicin (14.5 µg g^−1^ body weight, Cell Pharm, Bad Vilbel, Germany) as developed by our group [1, 3, 6]. Mice were kept in their normal cages to reduce distress after doxorubicin injection and proteinuria. Daily food and fluid intake were monitored by weighing the food pellets and the water bottle. Samples of spontaneously voided urine were collected in the morning between 8 and 10 am 2 days before (baseline) and up to 10 days following doxorubicin injection. Blood samples were drawn before induction and at sacrifice on day 10. In a subset of mice, sodium balance was studied in metabolic cages on a control diet (C1000, Altromin, Lage, Germany, sodium content 106 µmol g^−1^) before and on the 7th day after induction of nephrotic syndrome. In another subset of mice, ENaC activity was determined after intraperitoneal injection of vehicle and 10 µg g^−1^ amiloride in 5 µl g^−1^ injectable water and collection of urine for 6 h before and on the 7th day after induction of nephrotic syndrome. All animal experiments were conducted according to the National Institutes of Health Guide for the Care and Use of Laboratory Animals and the German law for the welfare of animals, and they were approved by local authorities (Regierungspraesidium Tuebingen, approval number M 7/18 G).

### Laboratory measurements

Urinary creatinine and plasma urea concentrations were measured with a colorimetric assay (Labor + Technik, Berlin, Germany), urinary protein concentration using the Bradford method (Bio-Rad Laboratories, Munich, Germany), and urinary sodium concentration with flame photometry (Eppendorf EFUX 5057, Hamburg, Germany). Both urinary protein and sodium concentration were normalized to the urinary creatinine concentration. Feces was dried, weighed, and dissolved in 1 M HNO_3_ before measuring sodium concentration using flame photometry. Plasma albumin concentrations were measured using a fluorometric kit against mouse albumin as standard (Active motif, Carlsbad, USA). Blood gas analysis was done using an IL GEM® Premier 3000 blood gas analyzer (Instrumentation Laboratory, Munich, Germany).

### Determination of FSAP antigen

Rabbit polyclonal FSAP antibody Ab (2 μg mL^−1^) was immobilized in microtitre plates and blocked with 3% bovine serum albumin (BSA). Diluted urine was added to the wells, and FSAP was detected by adding a monoclonal FSAP Ab (2 μg mL^−1^) followed by peroxidase couple secondary antibody. Absorbance was followed in a microplate reader EL 808 (BioTek Instruments, Winooski, OR, USA) at 450 nm. To quantify FSAP antigen in human urine samples, we use pooled standard human plasma as well as pooled normal healthy urine spiked with plasma-purified FSAP to generate standard curves and express activity as μg mL^−1^ [22]. Both standard curves gave similar results.

### Determination of FSAP activity

Microtiter plates were coated as described above for antigen assay. Diluted urine was added to the wells, and pro-uPA activation was measured by adding pro-uPA (1 μg mL^−1^; Saruplase, Grünenthal, Stolberg, Germany), and 0.2 mM of the chromogenic substrate S-2444 (L-pyroglutamyl-glycyl-L-arginine-p-nitroanilinedihydro-chloride) (Haemochrom Diagnostica, Essen, Germany). Absorbance was followed in a microplate reader EL 808 at 405 nm, and results are presented as mOD min^−1^.

### Western blot for FSAP expression in urinary samples

Human urine samples were separated by 10 or 12% SDS-PAGE under reducing or non-reducing conditions as indicated. Proteins were transferred onto PVDF membrane (Millipore, Billerica, MA, USA) and probed with primary antibodies which detect either FSAP zymogen at 64 kDa or the light chain at 27 kDa (Mab677) or the heavy chain at 50 kDa (Mab1189). Bound antibodies were detected using horseradish peroxidase conjugated secondary antibodies (Swine anti rabbit polyclonal; P0217, Dako, Glostrup, Denmark) and the enhanced chemiluminescence detection system (Amersham-Pharmacia, GE Healthcare, Freiburg, Germany). Murine urine samples from mice were separated by 8% SDS-PAGE under non-reducing conditions. Proteins were blotted onto a nitrocellulose membrane (Amersham, UK) and probed with a primary antibody detecting FSAP zymogen at 64 kDa (Mab570). Bound antibodies were detected with a fluorescent secondary antibody labeled with IRDye 800CW and a fluorescence scanner (Licor Odyssey, Lincoln, USA).

### Determination of the proteolytic activity of FSAP

Proteolytic activity was quantified using 20 µM of fluorogenic substrate Boc-Gln-Ala-Arg-AMC (Boc-QAR-AMC) (Boc: t-Butyloxycarbonyl; AMC: 7-Amino-4-methylcoumarin; R&D systems, Abingdon, UK) in a sample volume of 100 µL. This substrate detects the activity of a wide range of trypsin-like proteases. The experimental protocol was similar to that described in previous reports [14, 34]. The fluorescence signal resulting from substrate hydrolysis at the cell surface was continuously recorded over a time period up to 90 min using a TECAN plate reader (360 nm excitation/465 nm emission wavelength).

### Two-electrode voltage-clamp measurements using human ENaC expressing Xenopus laevis oocytes

Oocytes were collected from *Xenopus laevis* with approval of the animal welfare officer for the University of Erlangen-Nürnberg as described [13, 17, 23, 33, 37]. Defolliculated stage V–VI oocytes were injected with cRNA encoding human α-, β-, and γ-ENaC (0.2 ng of cRNA/subunit of human wild-type α-, β- and γ- or mutant γRKRK178AAAA ENaC). ENaC-mediated whole-cell currents were measured using the two-electrode voltage-clamp (TEVC) technique as previously described [13, 17, 18, 23]. Amiloride-sensitive current (Δ*I*_ami_) values were determined at a holding potential of − 60 mV by washing out amiloride (2 µM) with amiloride-free ND96-solution (96 mM NaCl, 2 mM KCl, 1.8 mM CaCl_2_, 1 mM MgCl_2_, 5 mM HEPES; pH 7.4 adjusted with Tris) and subtracting the whole-cell currents measured in the presence of amiloride from the corresponding whole-cell currents recorded in the absence of amiloride. To assess the stimulatory effect of FSAP on ENaC, we used recombinant FSAP serine protease domain (FSAP-SPD, 30 kDa), synthesized as described before [29], and determined Δ*I*_ami_ twice in a single oocyte (i.e. before and after exposure to the protease). After the first measurement of Δ*I*_ami_, the intracellular electrodes were withdrawn and the oocyte was placed for 5 min in ND96 to recover from the impalement and to reseal the plasma membrane [26]. Subsequently, the oocyte was transferred to 150 μL of protease-supplemented ND96, containing wild-type FSAP-SPD (FSAP-SPD-WT) or the inactive Marburg I variant of FSAP-SPD (FSAP-SPD-MI), or 150 µl of protease-free ND96 as a control. Unless stated otherwise, oocytes were incubated for 30 min before a second measurement of Δ*I*_ami_ was performed.

### Western blot from kidney tissue of mice

Western blot analysis of α-, β-, and γ-ENaC expression was performed from a membrane protein preparation of kidney cortex collected under control condition or on the 7th day after induction of nephrotic syndrome when urinary sodium retention concentration dropped below 20 mM, indicating maximal ENaC activation. Half the kidney per mouse was sliced, and the cortex was dissected using a scalpel. Homogenization was performed using a Dounce homogenizer in 1 ml lysis buffer containing 250 mM sucrose, 10 mM triethanolamine HCl, 1.6 mM ethanolamine, and 0.5 EDTA at pH 7.4 (all Sigma) [42]. During all preparation steps, aprotinin (40 µg ^−1^) and a protease inhibitor cocktail (final concentration 0.1 × stock; mini-complete, Roche) were present to avoid ENaC cleavage in vitro. Homogenates were centrifuged at 1000 g for removal of the nuclei. Subsequently, the supernatant was centrifuged at 20,000 g for 30 min at 4 °C, and the resulting pellet containing plasma membranes was resuspended and diluted to a concentration of 5 mg L^−1^. For analysis of γ-ENaC expression, samples were deglycosylated using PNGaseF according to the manufacturer´s instructions (NEB, Ipswich, USA) and as previously described [7, 16]. First, samples were denaturated with a glycoprotein denaturing buffer. Samples were then incubated with glycobuffer, NP-40, and PNGaseF for 1 h at 37 °C. For the analysis of α- and β-ENaC, native samples without deglycosylation were boiled in Laemmli buffer at 70 °C for 10 min. Subsequently, 20 µg of sample was loaded on an 8% (γ-ENaC) or 4^−1^5% (α- and β-ENaC) polyacrylamide gel for electrophoresis under reducing conditions. After transfer of the proteins onto a nitrocellulose membrane (Amersham, UK), the blocked blots were incubated with primary rabbit antibodies against murine α-, β- (custom-made, Pineda, Berlin), and γ-ENaC (SPC-403, Stressmarq, Viktoria, Canada) overnight at 4 °C after 1:1000 dilution in blocking buffer (Licor, Lincoln, USA) [4, 14, 28]. Antibodies were based on the peptide sequences described by Masilamani et al. [27] and validated using lysates from oocytes expressing murine αβγENaC against lysates from sham-injected oocytes as described previously [7]. Signals were detected using fluorescent secondary antibody labeled with IRDye 800CW or IRDye 680RD and a fluorescence scanner (Licor Odyssey, Lincoln, USA). After detection of α-ENaC, the membranes were stripped for detection of β-ENaC according to the manufacturer’s instruction using the NewBlot Nitro Stripping Buffer (Licor, Lincoln, USA). For loading control, total protein was measured using Revert Total Protein Stain (Licor, Lincoln, USA). For densitometry, ENaC signals were normalized for total protein signal of the entire lane using Image Studio version 3.1.4 and Empiria Studio version 1.3.0.83 (Licor, Lincoln, USA).

### Statistical analysis

Data are provided as arithmetic means with SEM. Data were tested for normality with the Kolmogorov–Smirnov test, D’Agostino and Pearson omnibus normality test, and Shapiro–Wilk test. Differences between healthy and nephrotic groups were tested for significance using *t*-test or Wilcoxon rank sum test. Densitometry from western blot was analyzed by a two-way ANOVA with state (healthy vs. nephrotic) and genotype (*Habp2*^+*/*+^ vs. *Habp2*^−/−^) as variables. Electrophysiological data were assessed by one-way ANOVA with Bonferroni post hoc test or paired *t*-test, as indicated in corresponding figure legends. A *p*-value < 0.05 was considered as statistically significant. Mouse and electrophysiological data were analyzed using GraphPad Prism (GraphPad Software, La Jolla, CA, USA, www.graphpad.com).

## Results

### Active FSAP is present in the urine of nephrotic patients and mice

**Fig. 1:**
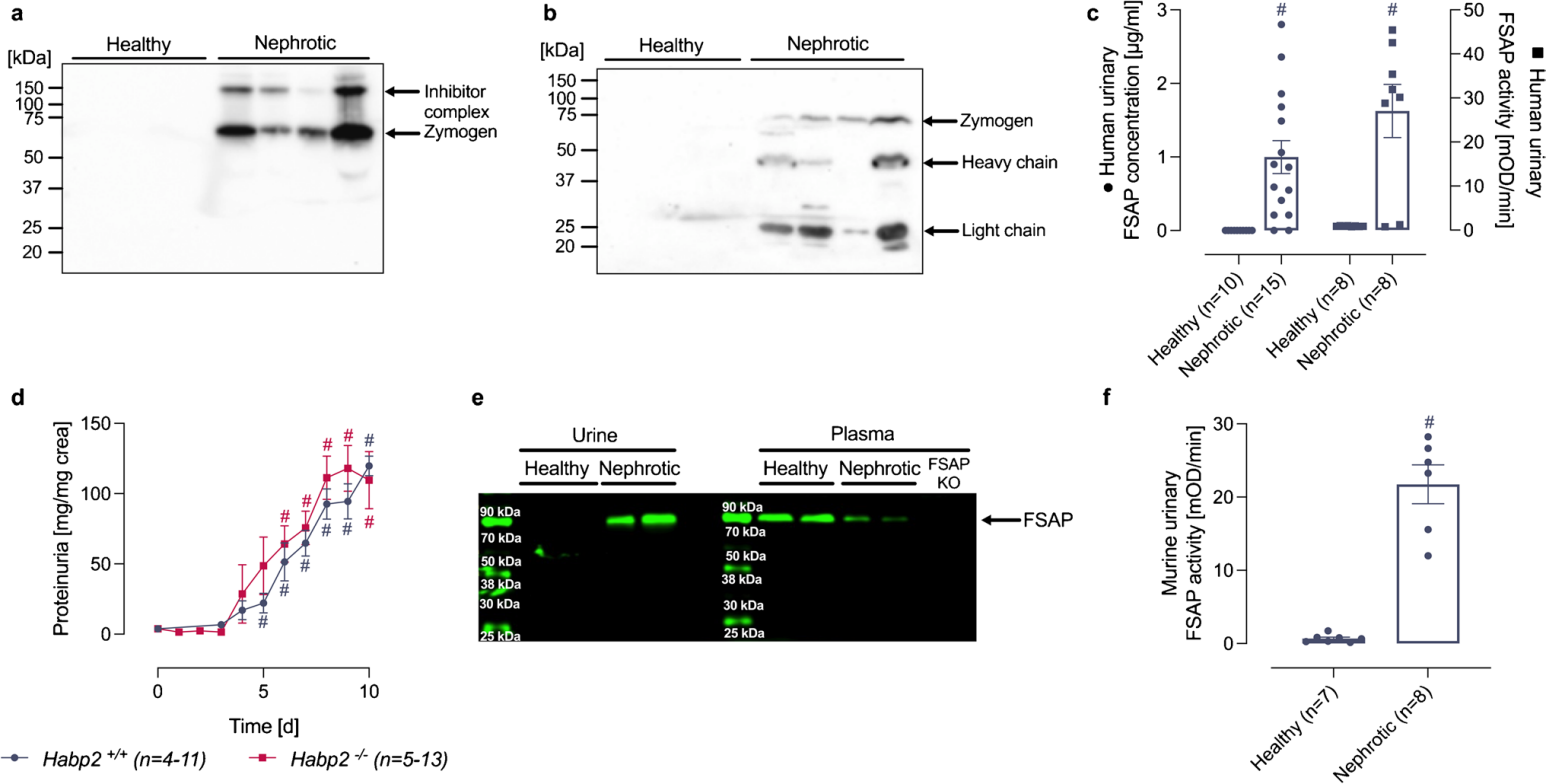
Urinary excretion of FSAP in nephrotic syndrome. **a** Western blot from human urine samples (*n* = 4 healthy, *n* = 4 nephrotic) under non-reducing conditions using a rabbit polyclonal antibody. In nephrotic samples, FSAP is detected at 64 kDa as zymogen and at 150 kDa as part of a inhibitor complex. **b** Western blot of the same samples (*n* = 4 healthy, *n* = 4 nephrotic) as used in (A) under reducing conditions using a mix of two monoclonal antibodies. In addition to the detection of FSAP zymogen as single chain (64 kDa), both the light (27 kDa) and heavy chain (50 kDa) are detected which requires previous cleavage at the activation bond R311. Both chains dissociate under reducing conditions. **c** Quantitation of urinary FSAP concentration and activity in human nephrotic urine samples. Activity was measured with pro-uPA as substrate after immunocapture of FSAP. Results are quantified as uPA chromogenic substrate turnover in mOD min^−1^. **d** Proteinuria in wildtype (*Habp2*^+*/*+^) and FSAP-deficient mice (*Habp2*^−/−^) after induction of experimental nephrotic syndrome by doxorubicin. **e** Western blot for FSAP expression from plasma and urine of *Habp2*^+*/*+^ mice (*n* = 2)*.* FSAP is detected at 64 kDa in its zymogen form in plasma samples and nephrotic urine. Compared to healthy plasma, FSAP expression appears to be reduced most likely due to urinary loss. The antibody does not recognize this band in the plasma from *Habp2*^−/−^ mice proving the specificity of the antibody. **f** FSAP activity in mouse urine from *Habp2*^+*/*+^ mice as determined with pro-uPA as substrate. Results are quantified as uPA chromogenic substrate turnover in mOD min^−1^. #Significant difference between healthy and nephrotic samples

In urine samples from healthy persons, FSAP was not detected by Western blot analysis under non-reducing conditions (Fig. 1a). In contrast, urine from nephrotic patients contained FSAP as single chain (64 kDa) representing the zymogen form as well as the FSAP inhibitor complex (~ 150 kDa; Fig. 1a). The identity of these SDS-stable protease inhibitor complexes was not investigated further. Under reducing conditions using two antibodies, FSAP was detected as the single chain zymogen form and in its two-chain form consisting of a light chain carrying the serine protease domain (27 kDa) and the heavy chain (50 kDa) (Fig. 1b). This finding indicates activation of FSAP by cleavage at the activation site, R311, and dissociation of both chains under reducing conditions. Proteolytic activation of FSAP was confirmed in an activity assay with pro-uPA as substrate, which detected active FSAP in nephrotic urine (Fig. 1c). Since this activity assay is based on the immunocapture of FSAP from the plasma, it is very specific for FSAP. The urinary concentration of FSAP in urine of nephrotic patients was variable and ranged from 0 to 2.8 µg mL^−1^ (Fig. 1c).

The presence of FSAP was also investigated in a mouse model of doxorubicin-induced nephrotic syndrome in *Hapb2*^+*/*+^ and *Hapb2*^−/−^ mice. Proteinuria was similar in both genotypes (Fig. 1d). In Western blot analysis of urine samples from healthy *Habp2*^+/+^ mice, FSAP was not detectable (Fig. 1e). In contrast, in urine samples from nephrotic *Habp2*^+/+^ mice, FSAP appeared with an identical size as in plasma (64 kDa; Fig. 1e). The antibody for detecting mouse FSAP is not capable of detecting reduced FSAP; hence, it was not possible to determine whether the FSAP was in a single chain or two-chain from. The plasma levels of FSAP were decreased in nephrotic mice which is consistent with its excretion in the urine. No FSAP was detected in *Habp2*^−/−^ mouse plasma (Fig. 1e). Using the activity assay with pro-uPA as substrate, active FSAP was detected in the urine of nephrotic *Habp2*^+*/*+^ mice (Fig. 1f). In conclusion, FSAP is present in human and murine nephrotic urine and is found in the active two-chain form.

### FSAP stimulates ENaC currents in Xenopus laevis oocytes expressing human ENaC by cleavage at the putative prostasin site

**Fig. 2:**
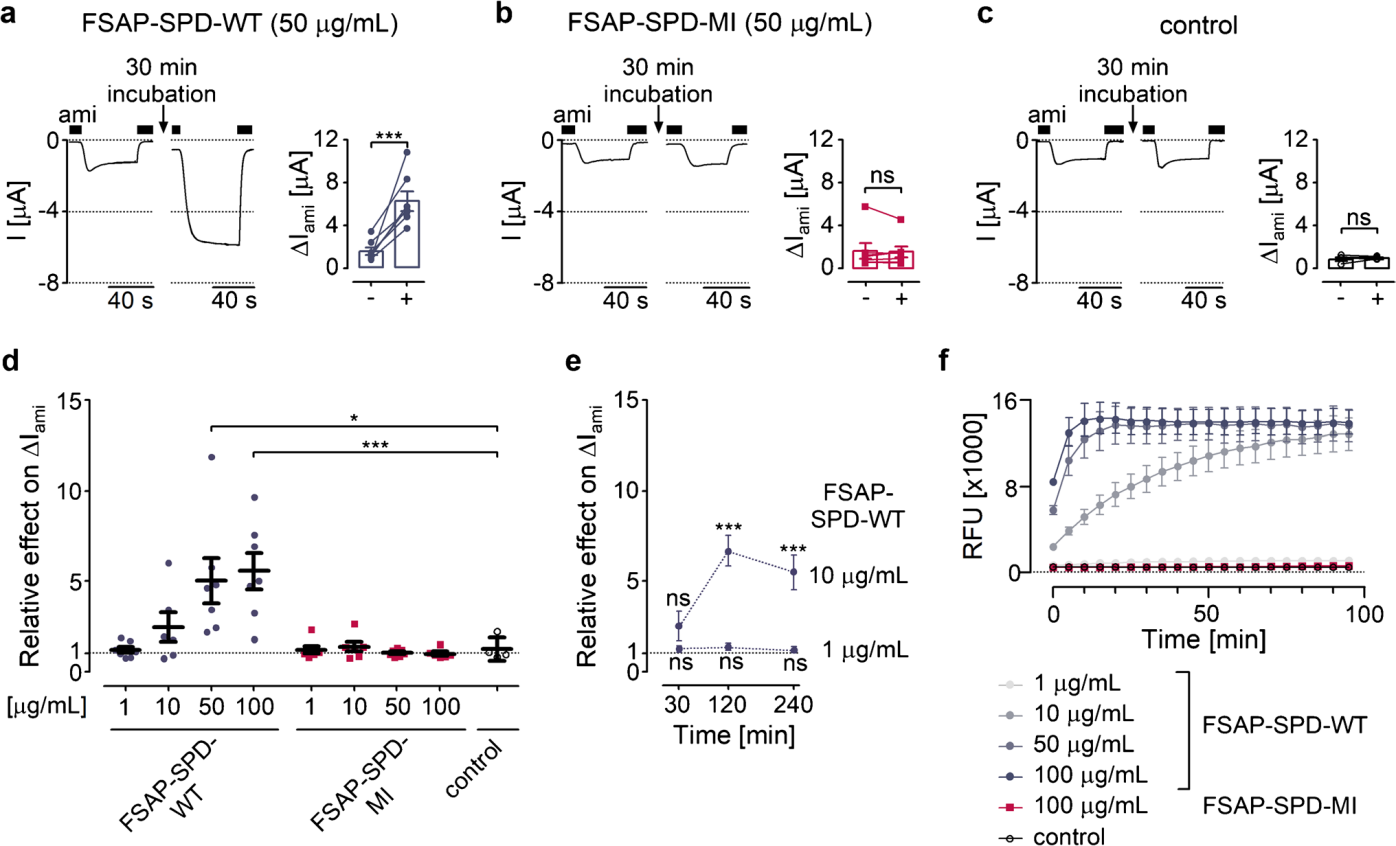
Stimulation of ENaC-mediated whole-cell currents by recombinant serine protease domain of FSAP. **a**–**c** Representative whole-cell current traces recorded in oocytes expressing human αβγENaC before (left panels) and after (middle panels) 30-min incubation in ND96 containing FSAP-SPD-WT (50 µg mL^−1^ or 1.67 µM; **a**) or FSAP-SPD-MI (50 µg mL^−1^; **b**) or in protease-free ND96 (control; **c**). Data obtained from similar experiments as shown in left and middle panels are summarized in corresponding right panels. Amiloride-sensitive currents (Δ*I*_ami_) were determined before ( −) and after ( +) incubation with FSAP-SPD-WT (**a**), FSAP-SPD-MI (**b**), or control solution (**c**). Measurements performed in the same oocyte are connected by a line (*n* = 4–7). **d** Summary of data from the same experiments as shown in **a**–**c** and from additional experiments in which different protease concentrations were used as indicated. Incubation time (30 min) was the same in all experiments. The relative effect on Δ*I*_ami_ was calculated for each oocyte as the ratio of Δ*I*_ami_ measured after and before the incubation period (*n* = 4–7). Each data point corresponds to one individual oocyte. **e** Effect of 1 (0.03 µM) or 10 µg mL^−1^ (0.33 µM) FSAP-SPD-WT on Δ*I*_ami_ for different incubation times (30, 120, or 240 min). Significance is indicated for comparison with baseline Δ*I*_ami_ measured before incubation with the protease (*n* = 5–7). **f** Time course of proteolytic activity in the indicated incubation solutions detected using the fluorogenic substrate Boc-QAR-AMC (RFU = relative fluorescence unit; *n* = 10–11). *p < 0.05; †*p* < 0.01; ns non-significant; paired *t*-test (**a**–**c**, **e**) or one-way ANOVA with Bonferroni post hoc test (**d**). Error bars, S.E

FSAP has a predisposition to cleave substrates with a cluster of basic amino acids [24]. Thus, FSAP cleavage sites show a remarkable similarity with previously identified cleavage sites in α- and γ-ENaC [10, 13, 21]. To study whether FSAP may stimulate sodium transport via proteolytic ENaC activation, we performed two-electrode voltage clamp measurements using *Xenopus laevis* oocytes heterologously expressing human αβγ-ENaC. The recombinant serine protease domain of FSAP (FSAP-SPD-WT; amino acids 292–560) has the same substrate specificity as full-length plasma FSAP [11, 29, 36]. Therefore, we used recombinant FSAP-SPD-WT for our in vitro experiments. As illustrated by representative current traces shown in Fig. 2a (left and middle panels) and summarized in Fig. 2a (right panel), incubation of ENaC expressing oocytes with FSAP-SPD-WT (50 µg mL^−1^) for 30 min strongly stimulated amiloride-sensitive whole-cell currents (Δ*I*_ami_). In contrast, Δ*I*_ami_ remained almost unchanged, when oocytes were incubated with the same concentration of a mutant FSAP-SPD carrying the inactive Marburg mutation (FSAP-SPD-MI; Fig. 2b). As expected, protease-free control solution had no significant effect on Δ*I*_ami_ (Fig. 2c).Δ

We also incubated oocytes with lower (1 and 10 µg mL^−1^) or higher (100 µg mL^−1^) concentrations of FSAP-SPD-WT or FSAP-SPD-MI. As shown in Fig. 2d, FSAP-SPD-WT stimulated ENaC in a concentration-dependent manner but with considerable variability between individual oocytes. With 100 µg mL^−1^, the average stimulatory effect was about fivefold and appeared to have reached a maximum. In contrast, FSAP-SPD-MI had no significant stimulatory effect on ENaC even when applied in a concentration of 100 µg mL^−1^. The stimulatory effect of proteases on ENaC currents is not only concentration-dependent but also time-dependent [18]. Indeed, when the incubation time was prolonged from 30 to 120 (or 240) min, the stimulatory effect of 10 µg mL^−1^ of FSAP-SPD-WT on Δ*I*_ami_ significantly increased and reached a similar level as observed with higher FSAP-SPD-WT concentrations (50 or 100 µg mL^−1^) and an incubation time of only 30 min (Fig. 2e). In contrast, no significant stimulation was observed with 1 µg mL^−1^ of FSAP-SPD-WT even after 240 min of incubation. The observed time- and concentration-dependence of the stimulatory effect of FSAP-SPD-WT on Δ*I*_ami_ corresponded well with the time- and concentration-dependence of its proteolytic activity measured using a fluorogenic substrate assay (Fig. 2f).

**Fig. 3:**
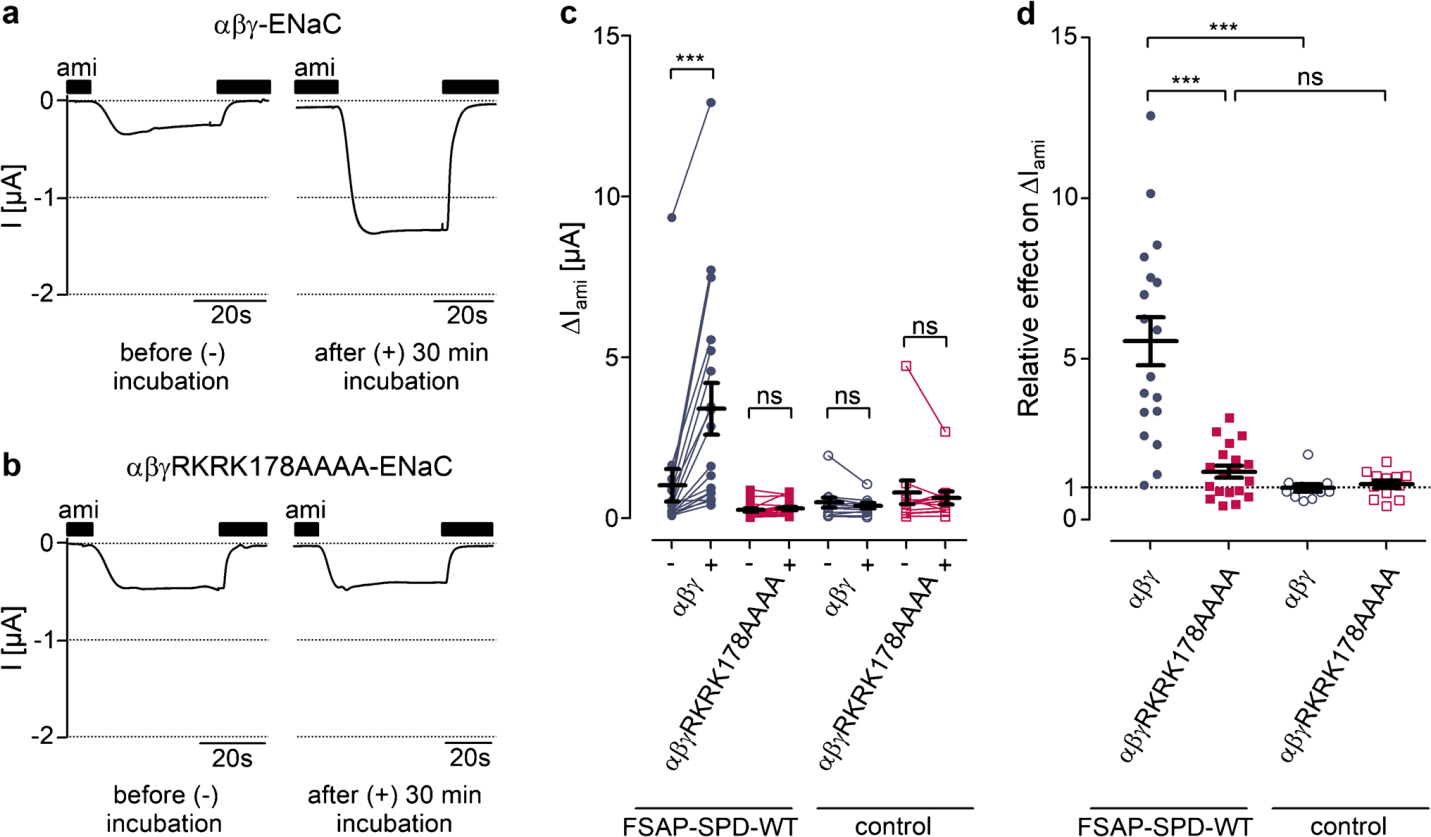
Mutation of ENaC at its putative prostasin cleavage site prevents its proteolytic activation by recombinant serine protease domain of FSAP. **a**, **b** Representative whole-cell current traces recorded in oocytes expressing human wild-type ENaC (αβγ-ENaC; **a**) or coexpressing wild-type α- and β-ENaC with mutant γ-ENaC (αβγRKRK178AAAA-ENaC; **b**) before (left panels) and after (right panels) 30 min incubation in a solution containing FSAP-SPD-WT (20 µg mL^−1^). **c** Summary of data obtained from similar experiments as shown in **a**, **b** and from additional experiments in which protease-free ND96 was used as control. Δ*I*_ami_ was determined before ( −) and after ( +) incubation in the indicated incubation solution. Measurements performed in the same oocyte are connected by a line (*N* = 2–3, *n* = 11–19). **d** Summary of the same data as shown in **c** normalized as relative effect of the indicated incubation solution on Δ*I*_ami_ (*N* = 2–3, *n* = 11–19). ‡*p* < 0.001; ns non-significant; paired *t*-test (**c**) or one-way ANOVA with Bonferroni post hoc test (**d**). Error bars, S.E. *N* indicates the number of different batches of oocytes; *n* indicates the numbers of individual oocytes measured

The stimulatory effect of FSAP on Δ*I*_ami_ is likely due to proteolytic ENaC activation involving the putative prostasin site in the γ-subunit of the channel as previously shown for other serine proteases like plasma kallikrein [19] and plasmin [18]. To identify the contribution of this site to the stimulatory effect of FSAP on ENaC, we co-expressed wild-type α- and β-ENaC together with a γ-ENaC subunit mutated at the putative prostasin cleavage site (γRKRK178AAAA). In parallel control experiments, we confirmed that wild-type ENaC was strongly stimulated by FSAP-SPD-WT by about fivefold (Fig. 3a, c, d). In contrast, in oocytes expressing the mutant ENaC, the effect of FSAP-SPD-WT on Δ*I*_ami_ was largely abolished (Fig. 3b, c, d) and not significantly different from that of protease-free control solution on wild-type or mutant ENaC (Fig. 3c, d). These data indicate that proteolytic ENaC activation by FSAP requires an intact prostasin cleavage site in the channel’s γ-subunit.

### Mice lacking FSAP (Habp2^−/−^) are not protected from sodium retention in experimental nephrotic syndrome.

**Fig. 4:**
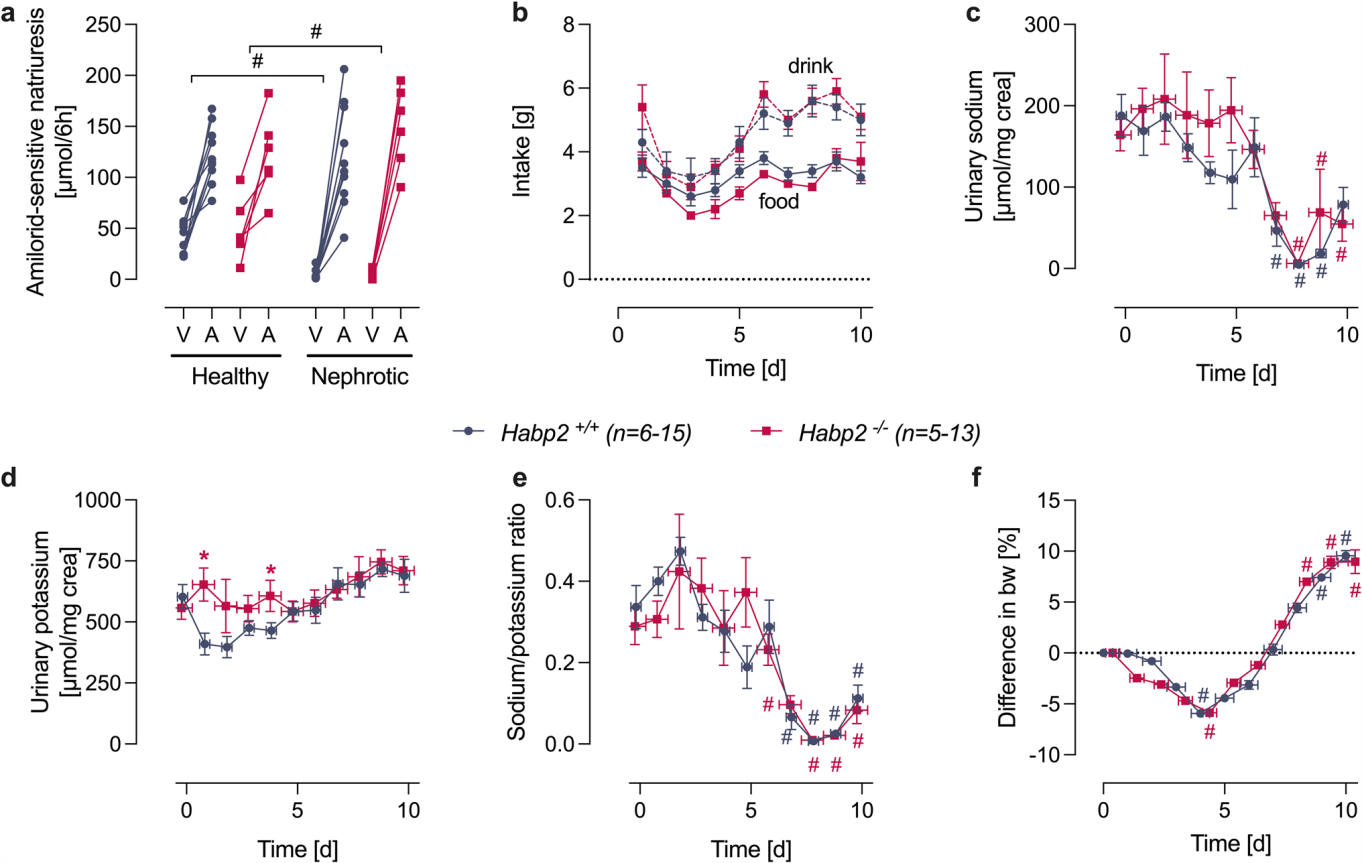
Impact of FSAP deficiency on ENaC activation and sodium retention in experimental nephrotic syndrome. **a** Natriuretic response to vehicle (injectable water, 5 µl g^−1^ bw) or amiloride (10 µg g^−1^ bw i.p.) in healthy and nephrotic *Habp2*^+*/*+^ and *Habp2*^−/−^ mice. Urine was collected for 6 h after injection and all mice underwent vehicle and amiloride injection sequentially (at day − 14/ − 13 and day 7/8, respectively). **b**–**f** Course of food and fluid intake, urinary sodium and potassium excretion and its ratio in spot urine samples and body weight taken in the morning after induction of nephrotic syndrome in *Habp2*^+*/*+^ and *Habp2*^−/−^ mice. Note: Due to a variance of one day in the onset of proteolytic ENaC activation in experimental nephrotic syndrome, the data in **c**–**e** were fit to the day of lowest urinary sodium (day 8) and to the day of lowest bodyweight (day 4) (**f**), which results in an x error depicted in the corresponding graphs. #Significant difference between healthy and nephrotic state. *Significant difference between the genotypes

To determine whether FSAP participates in ENaC-mediated sodium retention in vivo, we studied the course of experimental nephrotic syndrome in mice lacking FSAP (*Habp2*^−/−^) and their wildtype littermates (*Habp2*^+*/*+^)*.* Following doxorubicin injection, *Habp2*^−/−^ mice developed similar proteinuria (Fig. 1e) as *Habp2*^+*/*+^ mice. The natriuretic response to amiloride (10 µg·g bw^−1^ i.p.) was determined to assess ENaC activity in healthy *Habp2*^+*/*+^ and *Habp2*^−/−^ mice. Baseline natriuresis was determined from injection of vehicle (injectable water, 5 µl·g bw^−1^ i.p.). As shown in Fig. 4a, this response was similar in healthy *Habp2*^+*/*+^ and *Habp2*^−/−^ mice. The calculated ratio of natriuresis after vehicle and amiloride injection corresponding to the slope in Fig. 4a was 3.4 ± 0.6 and 4.0 ± 1.7 in healthy *Habp2*^+*/*+^ and *Habp2*^−/−^ mice, respectively, indicating similar ENaC function in both genotypes. After induction of nephrotic syndrome, this ratio increased significantly in both genotypes to 53 ± 13 and 46 ± 21 in nephrotic *Habp2*^+*/*+^ and *Habp2*^−/−^ mice, respectively, without a significant difference between the genotypes (*p* = 0.49). Studies of sodium balance revealed that *Habp2*^+*/*+^ and *Habp2*^−/−^ mice developed renal sodium retention after induction of nephrotic syndrome (Table 1). During the course of nephrotic syndrome daily urinary sodium concentration dropped to minimal values of 5 ± 1 µmol mg^−1^ creatinine and 6 ± 1 µmol mg^−1^ creatinine in *Habp2*^+*/*+^ and *Habp2*^−/−^ mice, respectively, despite constant food and fluid intake (Fig. 4b–c), urinary potassium excretion was not altered appreciably (Fig. 4d). Urinary sodium/potassium ratio was decreased in nephrotic mice of both genotypes indicating tubular sodium avidity (Fig. 4e). Subsequently, nephrotic mice gained body weight and developed ascites indicating sodium retention in both genotypes (Fig. 4f). The maximal body weight gain calculated from the difference of the body weight between day 4 and day 10 was 18 ± 1% and 21 ± 2% in *Habp2*^+*/*+^ and *Habp2*^−/−^ mice, respectively, which was not significantly different (*p* = 0.23).

**Table 1:** Sodium balance under a sodium replete control diet in *Habp2*^+/+^ and *Habp2*^−/−^ mice before and on day 7 of experimental nephrotic syndrome.

	Healthy		Nephrotic	
	*Habp2*^+/+^	*Habp2*^−/−^	*Habp2*^+/+^	*Habp2*^−/−^
Total Na^+^ intake, µmol 24 h^−1^	245 ± 26	265 ± 38	291 ± 38	261 ± 39
Urinary Na^+^ excretion, µmol 24 h^−1^	103 ± 22	108 ± 13	12 ± 4#	10 ± 3#
Fecal Na^+^ excretion, µmol 24 h^−1^	24 ± 4	37 ± 4*	13 ± 3#	11 ± 1#
Na^+^ balance, µmol 24 h^−1^	118 ± 22	120 ± 33	266 ± 36#	239 ± 37#

Arithmetic means ± SEM (*n* = 6 each). Note that the body weight was similar in *Habp2*^+/+^ and *Habp2*^−/−^ mice (25.6 ± 0.9 g vs. 24.7 ± 1.3 g, *p* = 0.59). #Significant difference between healthy and nephrotic mice; *significant difference between genotypes

Table 2 depicts the plasma concentrations of urea, albumin, electrolytes, hemoglobin, and pH. There were no significant differences in any parameter in healthy *Habp2*^+*/*+^ and *Habp2*^−/−^ mice. Plasma potassium concentrations were significantly increased compared to baseline in both genotypes. There was no difference between the genotypes (Table 2). In nephrotic mice, there was a marked decrease in plasma albumin concentration indicating negative albumin balance and a tendency towards increased plasma urea concentration pointing to a mild decrease of GFR. In nephrotic *Habp2*^+*/*+^ mice, plasma Na^+^ and hemoglobin concentration as well as hematocrit were decreased, consistent with dilution of the plasma volume. This finding was less prominent in nephrotic *Habp2*^−/−^ mice.

**Table 2:** Plasma parameters of healthy and nephrotic *Habp2*^+/+^ and *Habp2*^−/−^ mice before and on day 10 of experimental nephrotic syndrome.

	Healthy		Nephrotic	
	*Habp2*^+*/*+^	*Habp2*^−/−^	*Habp2*^+*/*+^	*Habp2*^−/−^
pH	7.24 ± 0.2	7.30 ± 0.01	7.29 ± 0.02	7.32 ± 0.02
std HCO_3_^−^, mM	22 ± 0.4	21 ± 1	24 ± 1	25 ± 2
Na^+^, mM	147 ± 0.4	147 ± 1	139 ± 2#	143 ± 2
K^+^, mM	4.4 ± 0.1	4.2. ± 0.1	5.8 ± 0.2#	5.7 ± 0.4#
Ca^++^, mM	1.09 ± 0.01	1.06 ± 0.02	1.11 ± 0.02	1.09 ± 0.05
Hct, %	44.3 ± 0.7	43.7 ± 0.8	37.2 ± 2.5#	43.7 ± 1.7
cHbc, g dL^−1^	14.6 ± 0.2	14.4 ± 0.2	12.3 ± 0.8	14.5 ± 0.6
urea, mg dL^−1^	51 ± 7	39 ± 3	58 ± 15	78 ± 28
albumin, g L^−1^	34 ± 2	35 ± 2	12 ± 1#	13 ± 2#

Arithmetic means ± SEM. Parameters from venous blood gas analysis were determined in n = 7–9 mice per group. Plasma urea and albumin concentration was determined in n = 4 mice per group. #Significant difference between healthy and nephrotic state; Abbreviations: std standard, Hct hematocrit, cHbc calculated hemoglobin concentration

### Expression of ENaC subunits and proteolytic activation of ENaC in nephrotic Habp2^+/+^ and Habp2^−/−^ mice.

**Fig. 5:**
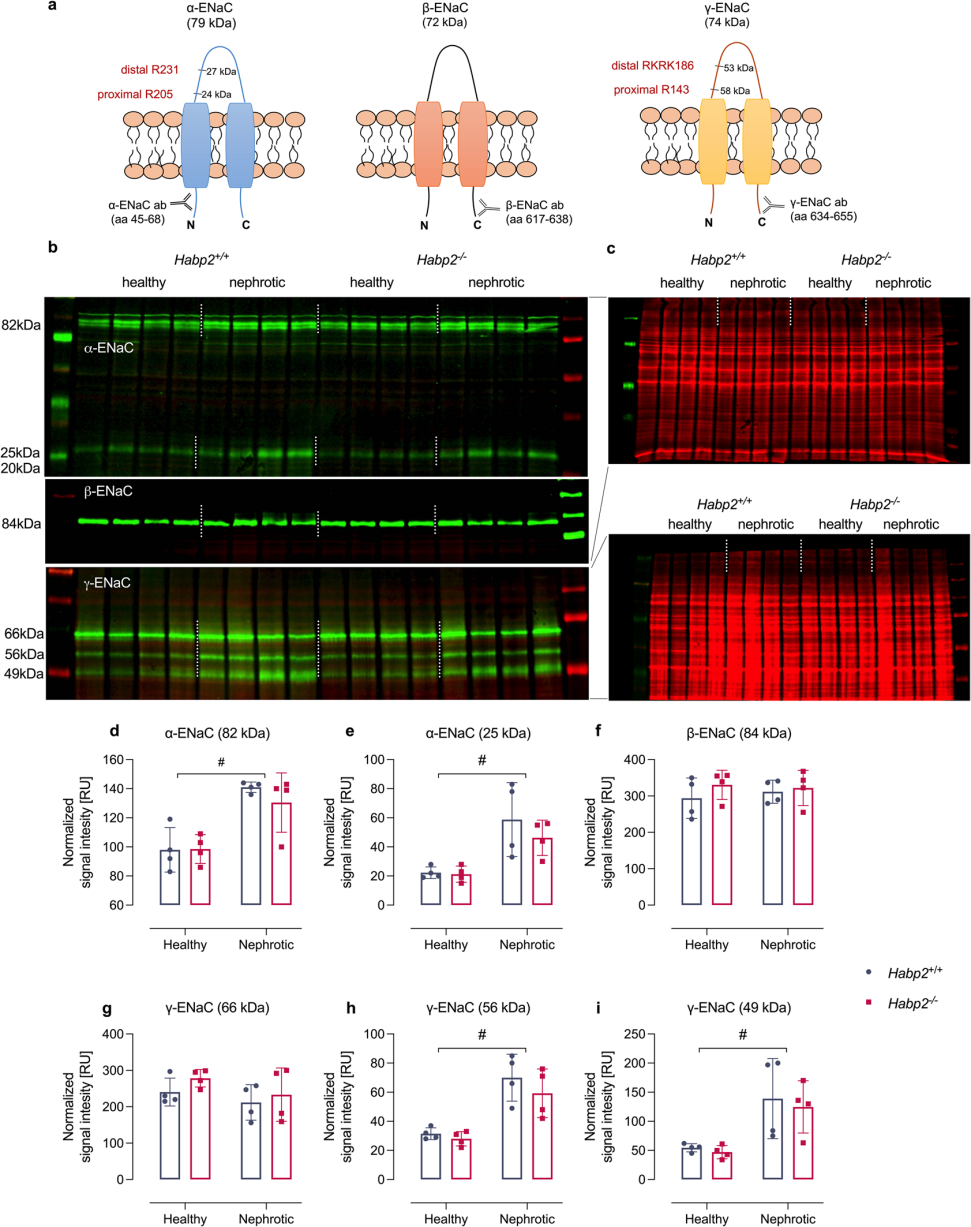
Renal expression of ENaC subunits in experimental nephrotic syndrome. **a** Localization of the immunogenic sequences of the used antibodies against murine α-, β-, and γ-ENaC. In α- and γ-ENaC, the proximal and distal cleavage sites (designated from the N-terminus, respectively) are depicted. The antibody against N-terminal α-ENaC is supposed to detect full-length α-ENaC at 79 kDa (699 aa) and two N-terminal fragments with a mass of 27 kDa (231 aa) and 24 kDa (205 aa). The antibody against C-terminal β-ENaC is supposed to detect full-length β-ENaC at 72 kDa (638 aa). The antibody against C-terminal γ-ENaC is supposed to detect full-length γ-ENaC at 74 kDa (655 aa) and C-terminal fragments with a mass of 58 kDa (512 aa) after proximal cleavage and at 53 kDa (469 aa) after distal cleavage, respectively. Mass values are calculated from the amino acid sequences (omitting any N-glycosylations). **b** Western blots showing the expression of ENaC subunits in a plasma membrane preparation of kidney cortex from *Habp2*^+*/*+^ and *Habp2*^−/−^ mice. α- and β-ENaC expression were analyzed in native samples on a 4–15% gradient gel after stripping, γ-ENaC expression was analyzed after deglycosylation of the same samples on an 8% gel. The higher molecular mass for α- and β-ENaC stems from N-glycosylation. **c** Total protein stain for control of loading and blotting. **d**–**i** Densitometry of the obtained bands normalized for total protein content of each lane (*n* = 4 each). ^#^Significant difference between healthy and nephrotic state (two-way ANOVA)

Western blot analyses from kidney cortex revealed two bands for α-ENaC at 82 and 25 kDa representing full-length and a cleavage product, most probably after distal cleavage (designated from the N-terminus; Fig. 5a, b). For γ-ENaC, three bands were detected in PNGase-treated samples at 66, 56, and 49 kDa representing full-length, proximally and distally cleaved fragments, respectively (Fig. 5c) [7, 16]. Specificity of these bands was confirmed by application of the immunogenic peptide and oocytes expressing murine αβγENaC as shown elsewhere [7]. For β-ENaC, there was only a single band at 84 kDa corresponding to the full-length subunit which is not proteolytically processed. In healthy *Habp2*^+*/*+^ and *Habp2*^−/−^ mice, there was no significant difference in the expression of any ENaC subunit. After induction of nephrotic syndrome, there was a significant increase of the expression of full-length α-ENaC, whereas the expression of full-length β- and γ-ENaC was not altered in both genotypes (Fig. 5d, f, g). In nephrotic *Habp2*^+*/*+^ and *Habp2*^−/−^ mice, the expression of the cleavage fragments of α-ENaC at 25 and of γ-ENaC at 56 and 49 kDa, respectively, were found to be significantly increased, indicating proteolytic ENaC activation at both subunits (Fig. 5e, h, i). However, there was no difference between the genotypes.

## Discussion

Our study reveals three novel findings: first, it demonstrates that FSAP is excreted in the urine of nephrotic patients and mice with kidney disease as a zymogen and as active protease. Secondly, FSAP was shown to stimulate ENaC currents in vitro through proteolysis of γ-ENaC at the putative prostasin cleavage site (γRKRK178). The stimulatory effect of FSAP on ENaC activity was prevented by mutating this cleavage site. Thirdly, nephrotic mice with FSAP deficiency were not protected from sodium retention and proteolytic ENaC activation. This latter finding indicates that FSAP is not essential for mediating sodium retention and that other serine proteases or mechanisms cause proteolytic ENaC activation and sodium retention in this model of nephrotic syndrome.

Interestingly, we detected active FSAP in urine samples of both nephrotic patients and mice in contrast to the plasma compartment where FSAP circulates as zymogen [41]. These concentrations were up to 25% of the plasma FSAP concentration of healthy humans. This raises the question how FSAP is activated in the tubule lumen after aberrant filtration. In the plasma, FSAP is mainly activated by free histones [41]. To date, there are not many studies that report the presence of histones in urine. In one study, histones related to the formation of neutrophil extracellular traps (NET) or NETosis were detected in the urine [43]. It is plausible that injury to the tubules causes the release of nucleosomes into the urine where the resident DNase cleaves the DNA to free the histones [32]. The involvement of specific urine-related factors in the activation of FSAP zymogen is also a possibility. Compared to plasma, nephrotic urine might contain lower levels of plasma protease inhibitors that will decrease the threshold for auto-activation of FSAP. Apart from cleavage of ENaC, there are other potential substrates of FSAP in the kidney tubules such as protease activated receptors [12]. These G-protein receptors could regulate other aspects of nephrotic syndrome, e.g., inflammation, which was not investigated in the current study [30].

To our knowledge, this is the first report that the serine protease FSAP activates ENaC. This effect was observed with 10 µg·mL^−1^ of the recombinant SPD domain of FSAP which is higher than the concentration of FSAP in nephrotic urine (up to 2.8 µg·mL^−1^). It should be noted that not all the recombinant protease is in an active form as we have reported before [29]. In mice, urinary FSAP concentration might easily exceed the concentrations observed in humans as mouse urine has a much higher solute concentration. For example, in doxorubicin-injected nephrotic mice, we found urinary plasminogen concentrations that were at least 100-fold higher than that found in nephrotic humans (> 100 µg mL^−1^ vs. 1 µg mL^−1^; [8, 37]). Therefore, FSAP concentration found in nephrotic urine might be sufficiently high to activate ENaC in vivo.

The results further indicate that FSAP activates ENaC by proteolytic cleavage of its γ-subunit at the putative prostasin site γRKRK178, which is important for proteolytic activation of ENaC [10, 13, 18, 19]. Mutation of this site abolished proteolytic activation by FSAP. The prostasin cleavage site is in perfect agreement of the substrate specificity of FSAP with a preference for basic amino acids R and K [24]. Previously, we reported that plasma kallikrein and plasmin can also activate ENaC by cleavage at the putative prostasin site [18, 19]. These findings underscore the importance of this cleavage site which represents a preferential site for trypsin-like serine proteases due to the polybasic amino acid sequence. In vivo, the decisive role of urinary serine proteases on ENaC-mediated volume retention has recently been shown by our group [8]. In that study, aprotinin treatment of nephrotic mice abolished volume retention in a similar way as treatment with amiloride. The effect of aprotinin was replicated in a genetic mouse model of nephrotic syndrome [40] and indicates that the therapeutic effect of aprotinin is mediated at least in part by preventing proteolytic ENaC activation [7]. So far, the identity of the serine protease(s) responsible for ENaC activation in nephrotic syndrome remains unknown. With the present study, we can exclude that FSAP is essential for stimulating sodium retention solely, at least in this mouse model for nephrotic syndrome. However, there is still the possibility that proteolytic ENaC activation may occur redundantly so that the lack of one will be compensated by another serine protease.

In conclusion, we show that FSAP is detected in the urine of nephrotic patients and mice and causes proteolytic activation of ENaC in vitro. However, this stimulatory effect of FSAP is not essential for sodium retention in nephrotic mice and is most likely mediated by other serine proteases present in nephrotic urine capable of proteolytically activating ENaC.

## Data Availability

The data that support the findings of this study are available from the corresponding author upon reasonable request.
